# The impact of adolescent suicide on professionals in secure residential youth care

**DOI:** 10.1080/0886571X.2023.2253724

**Published:** 2023-09-07

**Authors:** S.P.T. Kaijadoe, K.S. Nijhof, H. Klip, A. de Weerd, A. Popma, R.H.J. Scholte

**Affiliations:** a Karakter Child and Adolescent Psychiatry University Centre, Nijmegen, The Netherlands; b Pluryn, Nijmegen, The Netherlands; c Child and Adolescent Psychiatry & Psychosocial Care, Amsterdam UMC, Vrije Universiteit Amsterdam, Amsterdam, The Netherlands; d Behavioural Science Institute, Radboud University Nijmegen, Nijmegen, The Netherlands

**Keywords:** Adolescent suicide, impact, professionals, secure residential youth care, traumatic stress

## Abstract

Professionals in secure residential youth care (SRYC) in the Netherlands are regularly confronted with suicides of adolescents and can be referred to as secondary victims. However, little is known about the impact of suicides on these professionals. This study explores the impact of suicides on professionals working in SRYC. Semi-structured interviews (*n* = 14) were conducted with professionals from several SRYC institutions. A thematic analysis of the material yielded four main themes: i) impact of suicide on a personal level, ii) impact of suicide on a vocational level, iii) impact of suicide on professional responses, and iv) facilitators and barriers that facilitated or obstructed professional resilience and the prevention of future suicides. The experience of suicide is extremely distressing for professionals. Some interviewees were traumatized by the impact. The findings suggest that most professionals draw on coercive measures for other youngsters sooner and more often after being exposed to suicide. The most commonly reported reason for doing so was the fear of experiencing a fatal incident. Professionals in SRYC require skilled and dedicated support (postvention) following a suicide to minimize its detrimental effects on personal, professional, and team functioning. Further implications for daily practice and policymaking are discussed.

## Practical implications

- Create an organizational culture in which professionals feel supported to discuss fear and uncertainties in balancing safety and accountability when working with suicidal adolescents.
- Postvention is needed for professionals and teams exposed to suicide.
- Train and facilitate professionals in nonviolent resistance to prevent coercion and suicide.
- The need for more trained personnel and increased knowledge of suicide prevention is highlighted.
- The use of experiential knowledge from lived experience experts could be a valuable additional resource to improve care.

## Introduction

Each year, approximately 800,000 people die by suicide worldwide (World Health Organization, 2017). Although absolute numbers of suicide peaks among men aged 45–54 years old, the sharpest increase in the number of suicide deaths throughout the life span occurs between early adolescence and young adulthood (Cha et al., 2018; Nock et al., 2008). Whilst worldwide mortality rates for adolescents are decreasing, suicide rates for adolescents are rising, which makes suicide the leading cause of death among adolescents worldwide (CDC, 2020; Cha et al., 2018). In line with this trend, suicide is the leading cause of death amongst adolescents aged 10 to 20 years in the Netherlands, and these numbers are rising (Central Bureau for Statistics, 2020a). In 1970 the number of completed suicides among young people was approximately 1.6 suicides per 100,000, whereas this number has currently almost doubled to 3.1 suicides per 100,000 (Central Bureau for Statistics, 2020b). Nowadays, on average every week one adolescent commits suicide in the Netherlands (Central Bureau for Statistics, 2020b).

These increased suicide rates among youth effect professionals working in secure residential youth care (SRYC) who are increasingly confronted with suicidal behavior and suicides of residents (Inspectie Gezondheidszorg en Jeugd, 2018). In the Netherlands, on average, 2,000 adolescents aged 12–18 are treated in SRYC per year after a decision by the juvenile court (Jeugdzorg Nederland, 2020). Adolescents in SRYC have severe mental and behavioral health needs and are often considered a danger to themselves or their environment (Leloux-Opmeer et al., 2016; Weeland, 2017). The aim of SRYC treatment is to guarantee adolescents’ safety and prepare them to return to and participate in society. In SRYC, on average, 8–12 youths live together in groups supervised by trained group workers 24 hours per day; it must be noted that in recent years, there has been increasing attention paid to the development of family like and small-scale forms of residence. Among a range of professional responses, staff in SRYC may use interventions of last resort, such as restraint and seclusion, to manage the suicidal behavior of residents (Jeugdzorg Nederland, 2018).

Notwithstanding the increase of suicides among young people in the Netherlands (Central Bureau for Statistics, 2020b), there are currently no statistics available that will give us a reliable picture of the incidence or prevalence of suicides in SRYC over a longer period of time. An analysis of the figures collected by the Health and Youth Care Inspectorate in 2017 showed that of the 48 young people who committed suicide in 2016, 16 (33%) were treated in youth care. In 2017, 26 out of 81 adolescents who committed suicide (32%) were treated in youth care, 10 of them (12%) committed suicide in a youth care institution of which 6 suicides happened in SRYC (Central Bureau for Statistics, 2020b; Inspectie Gezondheidszorg en Jeugd, 2018). It also appeared that in 18 of the 26 cases, appropriate assistance for suicidal adolescents was lacking. In at least 16 of 26 cases there were problems in the cooperation between care organizations involved (Inspectie Gezondheidszorg en Jeugd, 2018).

In 2015 a large decentralization occurred in youth care, with which the Dutch government aimed to transform the youth care system. Given the high costs associated with placement in residential youth care, the transition of 2015 entailed budget cuts as well (Friele et al., 2019; NJI, 2019; Rijksoverheid, 2015). As a result of these budget cuts, youth mental health care clinics had to reduce their number of beds (Friele et al., 2019). Hence, young people with internalizing psychiatric problems such as depression, suicidal behavior and self-harm, were increasingly referred to SRYC facilities instead of being referred to youth mental health care clinics, as was the case in the past (Buysse et al., 2019). This is problematic as SRYC is historically developed for adolescents with externalizing problems (i.e., lying, no respect for others, verbal or physical aggression) (Vermaes et al., 2014). The theoretical framework of SRYC is rooted in pedagogical interventions, aiming at reduction of problem behaviors of youth, whereas in youth mental health clinics, the medical model (Diagnostic and Statistical Manual of Mental Disorders, DSM) is dominant (American Psychiatric Association, 2013). Professionals in SRYC feel insecure and indicate that they are not sufficiently trained to deal with adolescent suicidal behavior (Ministerie van Volksgezondheid Welzijn en Sport, 2019; Nijhof et al., 2017). Moreover, professionals are unable to successfully predict which adolescents will not only attempt but also complete suicide (Nijhof et al., 2017). Research by De Valk (2019) shows that professionals in SRYC tend to act more controlling toward residents than necessary (De Valk, 2019). In short, the increase of suicidal adolescents and completed suicides in SRYC is challenging for professionals working in SRYC (Buysse et al., 2019; Inspectie Gezondheidszorg en Jeugd, 2018).

Concerning the prevalence of professionals’ exposure to suicide, it is estimated that between 32–95% of professionals have been exposed to adult suicide (Lyra et al., 2021). More specifically, among psychiatrists, 81% experience the suicide of an adult patient at some point in their careers, and more than half consider leaving mental health care because of stressful working conditions (Bijlsma, 2012). Although suicide is the most likely cause of death for children and adolescents treated by psychiatrists (Al-Mateen et al., 2018), we did not find research on the impact of adolescent suicide on psychiatrists or mental health care professionals in general. Most research focuses on the impact of adult suicide on professionals in adult mental health care services (Lyra et al., 2021). Al-Mateen et al. (2018) attempted to review the literature on responses of clinicians to suicide of adolescents but found it lacking. Therefore, their article reviewed the topic of adolescent suicide and discussed the findings with literature concerning the impact of adult suicide on mental health clinicians (Al-Mateen et al., 2018).

What we do know, based on the literature on adult suicides, is that the impact on mental health professionals entails grief, guilt, self-blame, anger, fear, and self-doubt (Valente, 1994). Moreover, responses to adult patient suicide include shock, devastation, sadness, guilt, shame, and grief, as well as shock, panic, self-accusation, fear, and frustration, preoccupation and intrusive thoughts about the suicide (Chemtob et al., 1988; Hendin et al., 2000; Landers et al., 2010; Wang et al., 2016; Wurst et al., 2011). Changes in professional attitude contained heightened awareness of suicide risk, reduction in confidence, and more restrictive practices such as increasing the level of observation and detention (Landers et al., 2010; Wurst et al., 2011). The above mentioned effects are even stronger among professionals with less experience (Chemtob et al., 1988). The latter is alarming, as in the Netherlands, SRYC is burdened with a vicious cycle of high staff turnover rates, major cutbacks, high rates of sick leave, and a shortage of experienced and well trained personnel (Ministerie van Volksgezondheid Welzijn en Sport, 2019; Prismant, 2018). Research shows that professionals react much more intensely to inpatient suicide than to suicide committed at a patient’s home (Bultema, 1994; Gulfi et al., 2010). Although we could conceptually hypothesize that professionals in SRYC may suffer at least comparable impacts compared to professionals exposed to adult suicide, research on the subject is missing (Al-Mateen et al., 2018).

The lack of scientific knowledge is problematic as it limits the field’s understanding of the impact that suicide may have on professionals working in SRYC. As stated earlier, considering that professionals in SRYC are increasingly confronted with suicide of adolescents (Buysse et al., 2019; Inspectie Gezondheidszorg en Jeugd, 2018), it is important to understand how these tragic events influence professionals in SRYC on a professional and personal level and, ultimately, on their attitudes and responses toward suicidal residents. Therefore, further research on this topic is required. The current study aimed to explore the impact of suicide on professionals in SRYC and how it affects their subsequent responses to the suicidal behavior of adolescents in their care. To the best of our knowledge, this is the first qualitative study among professionals in SRYC that systematically explored this topic through the participants’ eyes.

## Method

### Study design and procedure

We conducted 14 semi-structured interviews to gain a deeper understanding of the impact of suicide on SRYC professionals. Interviews were analyzed using thematic analysis (Braun & Clarke, 2006). The study protocol was approved by the medical ethics review committee of Arnhem-Nijmegen (2019–5571). A mixture of purposive and convenience sampling was used (Patton, 2014). The interviews were conducted from October 2019 to December 2020. Interviews took place at the SRYC institute and lasted 1–2 hours. The interviews were conducted by a trained qualitative researcher (SK) and a lived experience expert. The trained lived experience experts (four females, one male) all had a social science degree (bachelor’s or master’s degree). Two child and adolescent psychiatrists (females) and two social workers (males), who all had experience with adolescent suicide, assisted in conducting one interview each. A semi-structured interview guide was used based on the following key themes: experiences with adolescent suicidal behavior, reactions toward suicidal behavior, experience with, and impact of suicide. The interview guide was used as a checklist. Each interview was conducted with a lived experience expert through open communication between the two interviewers and the participant. Given the sensitive nature of the subject, ample time was taken to get acquainted (“rapport”) before starting the interview (Patton, 2014). In addition, the lived experience experts briefly discussed their own experiences with the subject before the start of the interview. Open-ended and probing questions were asked to gain more detail on a particular issue. The interviews were audio-recorded, transcribed verbatim, and anonymized. Written informed consent was obtained from all the participants. In the member check, all participants agreed on the accuracy of the transcription. In two cases, professionals provided additional information about the transcript. The Consolidated Criteria for Reporting Qualitative Research (COREQ; Tong et al., 2007) was followed (see Supplementary table S1). Data saturation was achieved.

### Participants

Three SRYC institutes in the Netherlands agreed to participate in this study. The first author approached the participants via e-mail, phone, or face-to-face. Exclusion criteria included: i) interns and ii) temporary workers employed for less than a month. Eight females and six males between 22 and 63 years (average age 35 years), were included. All participants were white Dutch individuals. Participants had worked in SRYC between 2 and 30 years (average work experience was eight years). Eight participants worked as group workers (social workers and higher vocational education), six had specialist training in psychiatry (academic degree) and worked as psychiatrists or psychologists. All professionals had experienced adolescent suicide less than 24 months before the interviews, of which five were first responders.

### Description of analysis

Thematic analysis (TA) was used to provide a more detailed account of professionals’ narratives. The TA used in this study was obtained from Braun and Clarke (2006). TA is a process for encoding information, seeking patterns, and developing themes (Braun & Clarke, 2006). A theme is not necessarily dependent on quantifiable measures but can be understood as capturing something important about the data in relation to the research question and represents some level of patterned response or meaning across the dataset. We used an inductive, data-driven analysis, which implies that we did not start from an existing theoretical framework (Braun & Clarke, 2006). Transcripts were analyzed using Atlas.ti version 8.4 (Scientific Software Development GmbH, Berlin, Germany). All interviews were conducted in pairs: the first author, a lived experience expert, a child and adolescent psychiatrist, or a social worker. All five lived experience experts assisted the first author in conducting, transcribing, coding, and analyzing the interviews.

The analysis was carried out recursively, moving backward and forward between data and codes (Braun et al., 2019). After reading and re-reading to become familiar with the data, researchers started coding data segments relevant to answering the research question. Each interview was coded independently by two researchers (the first author and an experienced expert), followed by comparing codes and discussing differences to derive inter-coder reliability (Braun & Clarke, 2006). The use of multiple coders increased the diversity of data perspectives. While analyzing, the researchers had intense discussions and conversations with each other, in which the lived experience experts were an additional source of knowledge (Glaser, 2002; Glaser & Strauss, 2008). The next step of the analysis involved grouping data with the same issues in themes (Charmaz, 2014). The themes were then combined to construct overarching themes. The analysis was centered on the four main themes described in the Results section. The final stage of the analysis was to review the data to ensure that each theme was sufficiently supported and extract direct quotes illustrating the themes and subthemes. This analytical process was conducted at several research meetings. A research diary was maintained.

## Results

**Figure 1.**
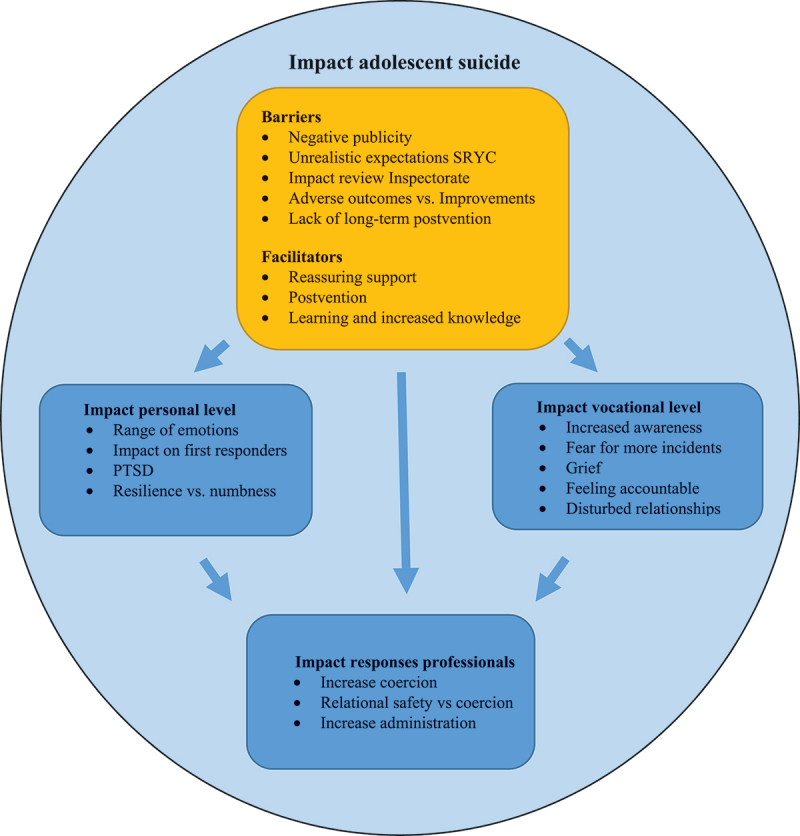
An overview of the relationships between themes

We conducted 14 semi-structured interviews to explore the impact of suicide on SRYC professionals. Results are organized across four main themes, which were strongly intertwined: i) impact personal level, ii) impact professional level, iii) impact on professionals’ responses to suicidal behavior, and iv) facilitators and barriers that facilitated or obstructed professional resilience and the prevention of future suicides. The personal impact of suicide refers to the emotional effects experienced by professionals in response to an adolescent suicide. However, while grief is a deeply personal emotion, it also significantly affects professionals’ vocational duties. Considering its relevance to the context of the study, “grief” is included in the vocational impact section. The impact of suicide on a vocational level entailed increased awareness, fear of more incidents, grief, feeling accountable, and disturbed relationships. The professional impact section delves into the implications of these vocational effects on the responses of professionals to adolescent suicidal behaviors. Themes are discussed separately below. The quotes were translated from Dutch. The relationships between themes are illustrated in Figure 1.

### Theme 1. Impact of suicide on a personal level

#### Range of emotions

In the narratives, a wide range of emotions was described in response to the suicide of an adolescent. The most commonly mentioned emotional responses were intense sadness, shock, guilt, self-doubt, feelings of failure, and powerlessness. For some professionals, their sadness was related to the death of the adolescent, while for others, it was associated with the sadness of a young person not being able to see a solution other than suicide. Some felt guilty and reflected that they should have prevented suicide completion. Hence, indirectly, they felt responsible for the tragic event. Some participants reported a fear of being blamed and held accountable. Others noted that having missed signals before a suicide added to feelings of distress and self-doubt, and shattered their confidence in their therapeutic abilities. Many participants spoke of an increase in feeling insecure. In summary, suicide had a major distressing effect on the emotional well-being of participants, as described in the following quote.

We see these young residents all day long, 24/7. I was on duty the day before it happened, and I did not notice the slightest thing in her behavior. This was a few hours prior to the suicide. Then you start doubting yourself, you actually think: “How could I have missed this? How is that possible?!” And then when this happens, it is devastating. Those feelings of failure and powerlessness are so overwhelming. (D15)

#### Impact on first responders

An additional, traumatic factor following death by suicide entails those who find the deceased, the so-called first responders. A total of five participants in this study were first responders. All reported emotional reactions including shock, confusion, and disbelief. Some participants described that these feelings became less significant over time, but their impact on daily life persisted over a more extended period. One professional explained how the sound of a helicopter caused her to panic, as it reminded her of a suicide in which an air ambulance had been deployed. Although the tragic event occurred almost a year ago, it still affected her daily life. Another participant described how he was unprepared and overwhelmed when finding the body of an adolescent who hung herself. He had to cut the youngster loose and provide first aid and CPR, but the girl died. After returning home, he broke down. This tragic event affected his daily life for several months.

I came home and opened the bathroom door and saw these washing lines hanging. They freaked me out reminding me of what had happened a few hours earlier. I broke down, I cried, and kept thinking “What is happening here?” For many months I did not dare to open up a door at home and at work. (D16)

#### Posttraumatic Stress Disorder (PTSD)

Some participants reported symptoms of depression and anxiety over a longer period after experiencing a client suicide. For two participants, these symptoms developed into PTSD. One participant described how experiencing an adolescent’s suicide changed his attitude as a person and professional. In the interview, he told how he had to call in sick. The impact of this event resulted in high levels of impairment in his daily activities at home. Hence, he underwent trauma therapy for recovery.

“I have experienced a completed suicide. I found her. [Short silence] And … … .well. Uhm. [Short silence] … I had to follow EMDR, I was diagnosed with PTSD, and I had to stay home for months. I could not do anything anymore. Nothing. I did not even dare take my children to swimming lessons. That is how scared I was. I thought: “I cannot do this job anymore, I do not want this anymore, I have failed.” (D39)

#### Resilience versus numbness

In addition, some participants shared that a completed suicide and repeated exposure to suicidal incidents diminished their level of responsive reactions and caused feelings of numbness. One participant shared how he had become numb to the suicidal utterances of adolescents because he had heard those stories too many times over the years. As expressed by many professionals, he also believed that suicide could not be prevented or predicted. Experiencing suicide confirmed this belief and made it easier to reconcile with suicide. Others described how, out of fear of not being able to return ever again, they forced themselves to return to work as soon as possible after a suicide. The group worker in the following quote displayed much resilience in his work after exposure to a suicide attempt, and two completed suicides.

I experienced one attempt, in which I could save her, so that ended well. And two fatal suicides. However I still love being able to help these young people with complex and difficult behavioral problems. It is meaningful work. Really, it is the most beautiful job in the world. (D33)

### Theme 2. Impact of suicide on a vocational level

#### Increased awareness of suicide risks

All professionals reported an increased awareness of suicide risk after experiencing suicide. Moreover, because of this awareness, the majority started using suicide risk assessments more often. They did so to try to predict suicidal outcomes, even though the majority of professionals held the opinion that even by using a formal suicide risk assessment, one cannot prevent or predict suicide. In their experience, adolescents serious about committing suicide were reluctant to express their intentions. Moreover, the value of a risk assessment relies upon adolescents to be forthright about their feelings and intentions, which, according to professionals, is seldom the case.

If someone has an active death wish, they can say to your face that things are going really well. And ten minutes later he can commit suicide. So you can never make a realistic estimate of the risk. (D15)

#### Increased fear of fatal incidents after suicide

Considering the increased awareness of the limitations of predicting suicide, some participants expressed increased fear after experiencing suicide. Knowing that suicide is a reality increases fear of working with suicidal adolescents. Participants described that witnessing suicide was overwhelming and often triggered strong emotional reactions in surviving group members and other residents who knew the deceased. Hence, an important item of concern to professionals in the event of suicide is to minimize and contain group contagion. Notwithstanding these efforts to prevent group contagion, experiencing suicide often results in an increase in suicidal behavior among the group members of the deceased. However, for some professionals, this increase in suicidal behavior reinforced their fear of fatal incidents. In the words of one professional,

We have had several suicide attempts and two successful suicides in the past year. That is reality. When this happens, there is a lot of distress, and some of the children become more suicidal. So as a group worker there is always this fear when you open a door that there is a child hanging there. (D2)

#### Grief after the loss of an adolescent

All the participants reported feelings of grief. Some participants became visibly emotional while describing their feelings of profound sadness during the interviews. Others recalled how they had to shut off their own emotions to be able to act in the acute crisis situation in the hours after the suicide. Participants described being touched by the grief and intense sadness of group members while coping with their own feelings. For some participants, managing their feelings of grief over the loss of an adolescent was too difficult while having to take care of the group members of the deceased. Feelings of grief were common, but the intensity of grief seemed to depend on the kind of relationship the professional had with the deceased. Moreover, some participants felt that their relationship with the deceased was intense as they had daily contact with them at the time of death. Hence, emotional closeness and perceived responsibility toward adolescents seem to have an important effect on the emotional impact of suicide.

I have experienced two suicides up until now. With the first I was more emotionally involved than the second. We had a strong therapeutic relationship. The more she got into crisis, the more genuine were the feelings we shared. I would see her again within a few days, but she made an attempt. It was one of many attempts, only this one was fatal. It had a huge impact on me. (D1)

#### Feeling accountable

The majority of participants felt accountable for the adolescents in their care and emphasized that the goal of admission in SRYC is to provide safety. Consequently, some professionals have struggled with the painful reality of a young person in their care dying by suicide. As a result, some felt guilty for being unable to keep the deceased safe or prevent suicide. Conversely, many participants held the opinion that suicide could not be prevented. For some professionals, the proclaimed aim of zero suicide in society was perceived as impossible to achieve. Moreover, some perceived the zero-suicide movement as negative, as it increased anxiety and self-blame in the aftermath of suicide. When a suicide is preventable, it implies that someone should be held accountable. This belief makes it harder to reconcile with the loss. Even professionals who could not be held accountable, given their hierarchical function, felt more or less accountable for the suicide. A group worker explained:

The last suicide was difficult to let go off. I dreamt about it. However, at a certain point I tried to let it go. I really do everything I can to …. to prevent it, only. Everything cannot always be prevented. I am over 100% committed, though, only I can and should not blame myself if it does happen. But you do. (D4)

#### Disturbed relationships

Some participants described how the suicide of an adolescent had multiple consequences for relationships among team members, as well as management. In some cases, relationships were disturbed after a suicide, and tension arose in the organization. One participant described how the management would respond to calls for accountability without paying attention to the impact of the tragic event on the professionals involved. Negative judgments and the fear of being blamed resulted in a tense atmosphere. In some cases, this resulted in teams falling apart. The participant in the next quote was visibly disappointed that problems in her organization had not been taken seriously in the aftermath of the suicide. One year after the fatal incident, she still had to work with inexperienced and insufficiently trained staff. Turnover rates were high, and new employees were hired, but left their new jobs very quickly out of dissatisfaction with the high workload and manpower shortages. Finally, she decided to resign as well.

I just don’t agree with how the organization handled things after the suicide. These are my last days here; I will be gone the next month. Since the suicide there has been little improvement in the care offered. There is no support or supervision. I am the last one working here; everyone else of my team has already left due to the bad working circumstances. (D15)

### Theme 3. Impact of suicide on responses of professionals

#### Increased coercion

Professionals stressed that if they had concerns about the safety of an adolescent, they were inclined to take coercive measures to provide safety sooner after experiencing a suicide than they would have done before the tragic experience. The most commonly reported reason for doing so was fear of more fatal incidents. However, running through the concept of coercion out of fear, most professionals underlined not having enough time and resources to enhance their relationships with adolescents, which increased feelings of fear. Professionals talk in order to make contact with suicidal adolescents and assess suicide risks accordingly. However, in the end, they have to decide whether the adolescent is safe or if the adolescent should be protected against himself, and control should be taken over. Experiencing suicide was often a reason to opt for caution. Hence, out of fear of more incidents, most professionals decided to take no risks and opted for physical safety by using coercive measures in response to adolescent suicidality. A group worker described his considerations as follows.

We experienced two suicides in a short period of time. Ever since I do not take any chance. If someone behaves suicidal, and I do not trust it, I will put him or her in isolation to keep them safe. Cause in the end I am the one who opens the door the next morning. (D33)

In case of concerns regarding a suicidal adolescent, the group workers consult the psychologist responsible for the treatment policy. Together they estimate the suicide risk and determine the appropriate treatment policy. In doing so, professionals tended to act more cautiously in the aftermath of a suicide, which resulted in an increase in consulting colleagues. Even in the process of deciding together, most professionals reported increased use of coercive measures in response to suicidal behavior. The most frequently used coercive measures were involuntary seclusion, restraint, removal of means, and camera observation. In the next quote, a professional described how her decision to use solitary confinement was influenced by her experience of suicide.

For example, we just had a completed suicide here, I really had to think twice when I had to make a suicide risk assessment. I noticed that all of a sudden, I chose safety, above all. Even though the young person might have been able to handle more autonomy, I still opted for seclusion. So in my case, experiencing a completed suicide really influenced my subsequent decisions. (D4)

#### Relational safety versus coercion

It is important to note that a few professionals advocate the importance of offering relational safety, even after experiencing suicide. According to them, developing a supportive relationship in which a bond of trust played an important role was crucial for adolescents to overcome suicidality. Notably, in full awareness of the risks, these professionals did not increase the use of coercive measures after a suicide. Moreover, they emphasized the importance of connectedness and relational safety in response to suicidal behavior. One of these professionals described working closely with colleagues in a team where she could discuss her fears and doubts. This helped her avoid using coercion in response to suicidal behavior. Therefore, however compelling, a minority of professionals resisted the increased demands for physical safety after a suicide and provided relational safety instead.

There was a lot of panic. The management was afraid and started saying, “Oh no, it cannot happen again and if we do not do it right then we are responsible for it.” But you know, we must ensure that we act carefully and can account for our actions, without acting more coercively and controlling. I had this discussion about safety up to the Executive Board. But I did not give in. (D5)

#### Administration

A common and frequently mentioned response to suicide was administrating files in a more precise manner. Out of fear of being blamed and held accountable for the death of an adolescent, participants felt an increased need for justification and registration. Depending on their hierarchical function, some professionals feared facing disciplinary penalties. Other participants spoke of a culture of blame, which referred to being held accountable for a suicide. Increased caution was reflected in an increase in the time spent administrating. In doing so, professionals felt more reassured and capable of justifying in hindsight the actions taken in the event of a fatal incident. However, spending more time administrating had various adverse side effects.

In the event of a suicide you have to be able to show them that you have acted carefully. So, you want to have what you have done on paper. However, the increase in reporting can have counterproductive effects. People become more rigid in following rules. More time administrating means there is less time left for contact with young people who are suicidal. (D1)

### Theme 4. Barriers and facilitators

The interviews revealed barriers and facilitators in terms of professional resilience and the prevention of future suicides.

### Barriers

#### Negative publicity

The suicide of an adolescent had a significant impact on the public image of the SRYC facility. Participants stressed that the negative publicity surrounding incidents in SRYC caused distress and added to a culture of blame. Professionals referred to the culture of blame as being held accountable for the failure of SRYC to help and cure suicidal adolescents. Hence, many participants perceived negative media attention as a barrier to overcoming feelings of guilt and self-doubt after a suicide.

The role of the media is very negative, which has an obstructive effect. The condemnation and blame-seeking are paralyzing and do not do the sector justice. Feelings of guilt and fear surrounding suicides are reinforced by negative media publicity. (D1)

#### Unrealistic expectations of SRYC

According to participants, the general public imposed very high expectations on SRYC as a sector, which was perceived as a barrier. Some professionals indicated that the public’s high expectations were partially informed by an erroneous belief that professionals in SRYC can predict which adolescents are at risk and then manage these risks in such a way as to prevent suicide. In addition, most participants indicated that during the past years, they had witnessed an upsurge of adolescents admitted to SRYC, wherein suicide risk played a significant role in their psychopathology. At the same time, professionals underlined that the SRYC field faced insufficient resources, such as budget cuts, lack of well-trained personnel, and high turnover of personnel, which prevented the efficacy of SRYC.

We seem to be more and more some kind of a last resort for the most complex youth. Therefore if no one knows anymore, they turn to us and expect us to solve it. We see girls aged 16 and 17 years who have been through so much that things got out of hand completely. They stay with us for six months, and everyone expects that we will fix it by then. But we do not have some kind of wonder pill! Of course we can’t fix that. (D33)

In addition, some professionals indicated that due to the lack of time and sufficient manpower, SRYC was not an appropriate place to treat suicidal adolescents effectively. Others expressed that taking into account the long waiting lists in youth mental health care, SRYC was the best alternative for suicidal adolescents. In short, professionals underlined that given these limitations, a more realistic expectation pattern would help alleviate expectations that are too high. According to them, SRYC cannot guarantee to keep an adolescent 100% safe, which became poignantly clear after a suicide.

Because [the name of the deceased] had no … ., there was no visible death wish; [name of the deceased] never made any statements in that sense. We never, never thought, that [the name of the deceased] would do this. We all were shocked and puzzling. Left with one question: Why? (D15)

#### Health and youth care inspectorate

Another frequently mentioned barrier entailed the review procedure of the Health and Youth Care Inspectorate. After a suicide, the Inspectorate reviews the files and documentation used in clinical practice, and professionals must substantiate how they acted. Professionals were asked to elaborate on which risk factors were controlled for, where, when, by whom, and why. Consequently, some felt that this search for failures of risk management was a search for someone who could be pinpointed as responsible for the suicide. They advocated that suicide reviews focus on issues reported in files close to the time of the suicide. However, adolescents in SRYC often have a long history in youth care, which is not included in the Health and Youth Care Inspectorate review procedure. Moreover, reviews of hindsight fallacies implied that an improved risk assessment could have led to a different outcome. As a result, some professionals felt blamed for inadequate recognition of suicide risk. For some professionals, the review procedure of the Inspectorate increased feelings of guilt.

They look in your files and question the choices you made. And looking back it is very easy to say: Yes, that did not go well, and this was not correctly estimated. And indirectly, they put the blame on you and you end up feeling guilty. (D16)

#### Adverse outcomes versus improvements

The investigation procedure of the Health and Youth Care Inspectorate often contributed to adverse outcomes, such as the previously described increase in record keeping, increased demands for safety, and increasingly restrictive practices. Conversely, a few participants reported that improvements in the quality of care were realized due to compelling recommendations of the Inspectorate. Examples included staff completing suicide prevention training or ameliorations in the transfer and collaboration between youth care institutions that would otherwise not have been installed on such a short notice.

We’ve had three suicides in a year and a half. The Inspectorate came, and we all had to follow a suicide prevention training. We have learned a lot from these suicides as well as from the suicide-prevention training we had to follow. (D5)

#### Lack of long-term postvention

Postvention refers to the actions or interventions taken after a traumatic event has occurred, particularly to support individuals affected by the event.

Postvention was perceived as both a barrier and a facilitator. Participants described a lack of long-term help and support after a suicide. In most cases, the initial experience of compassion and support from colleagues and management disappeared after a few weeks. In general, participants noted that attention paid to the impact of suicide decreased after a few weeks. Even less support and awareness was provided after a few months. In the long term, participants reported a lack of communication, a perceived taboo on talking about suicide, and a lack of support from management.

When I look at my own situation, a year after the suicide, there was no one within the organization who asked me how I was doing. No one. I decided to arrange a meeting with colleagues who worked on the night of the suicide. We had dinner together to commemorate her death. So, a year after the suicide, there is no attention at all to what happened. No, it rather is a taboo to talk about it. (D39)

### Facilitators

#### Reassuring support

Positive, reassuring support from management and colleagues was described as an essential facilitator in the aftermath of a suicide. The coping strategies most helpful were talking to colleagues in an open and safe team climate. However, according to most participants, this beneficial attention was, in many cases, not sufficiently long. Sharing personal feelings in a non-judgmental atmosphere, as well as supervision and informal chats, were described as facilitators and helpful support, but became less frequent over time due to high work demands, high turnover rates, and a perceived taboo on discussing the impact of suicide, resulting in silencing the subject. Not being exposed to negative judgments from management and colleagues is also a helpful resource for professionals.

What I really appreciated was that management didn’t immediately put the blame on anyone. We also felt supported by the management towards parents. Instead of being judged, because that is not what you want, they actually supported us. That really went well, and for me that was very important. (D22)

#### Short-term postvention

Most participants spoke of well-organized postvention for peers and the team immediately after the suicide. Short-term postvention included group and individual talks with adolescents and professionals. Memorials and church services were often organized after a suicide. A few participants described how mourning with group members was helpful in their grieving process. Mourning included being able to reminisce about the deceased (in groups and individually) by writing farewell messages or going to the room where the tragedy occurred and visiting the deceased’s grave. These forms of support and attention for peers and professionals shortly after the tragedy were perceived as helping to cope with the impact of the suicide.

There was a lot of team support. I took care of a colleague who had performed CPR on the diseased. There was also much support for the group; the doors of all the children were left open at night, and a group leader was present at the hallway throughout the night. That went really well. (D33)

#### Learning and increased knowledge

A few professionals reported that discussing the suicide in retrospect with colleagues and supervisors was perceived as helpful and enabled professionals to learn from the tragic experience. An open, safe working climate was an important facilitator of learning from a suicide. However, several participants advocated that it would have been more beneficial if this period of intense attention had lasted longer after the tragedy. In their opinion, the daily routine took its course too soon. Furthermore, a commonly mentioned facilitator involved consulting other colleagues more often in the case of adolescent suicidal behavior. After receiving suicide prevention training following a suicide, professionals reported an increase in knowledge of suicide prevention, which facilitated discussions on suicidal behavior in adolescents. Therefore, an increase in attention paid to suicide can improve daily practice and facilitate learning.

Yes, I have really seen tremendous progress among my colleagues in sharing and talking about suicidal behavior of adolescents and how it impacts them. We have learned in the aftermath of the three suicides we experienced here. (D5)

## Discussion

This study explored the impact of suicide on professionals working in SRYC units. Furthermore, we examined how this impact affected the subsequent responses of professionals to suicidal adolescents. The main findings of this study were twofold. Firstly, most professionals experience high levels of distress after experiencing suicide. Secondly, the majority of interviewees reported using coercive measures sooner and more often in response to suicidal behavior after exposure to suicide. The main reason for doing so was the fear of experiencing a fatal incident again. Moreover, the interviews revealed barriers and facilitators that facilitated or obstructed professional resilience and the prevention of future suicides. To the best of our knowledge, this is the first qualitative study among professionals in SRYC that provides a rich systematic exploration of this topic using participants’ first-person accounts. As we did not find comparative research on the impact of adolescent suicide on health care professionals, comparisons of our results with existing research in this field were not possible. To the best of our knowledge, this is the first qualitative study among professionals in SRYC that provides a rich systematic exploration of this topic from participants’ first-person accounts. We discuss the main results in more detail below.

### Coercive cycle

The results revealed an increase in restrictive practices in response to the suicidal behavior of adolescents after exposure to suicide. The perceived increase in restrictive practices is problematic because research reveals that coercion has negative consequences for youths (Fisher, 1994; Haugom et al., 2019; Knox & Holloman, 2012; LeBel et al., 2010; Linke et al., 2002; Roy et al., 2021) and professionals (Goulet & Larue, 2017). In line with research, professionals have reported that suicide often increases suicidal behavior of the group members of the deceased (Kaijadoe et al., 2023). The increase in suicidal behavior of group members adds to the already elevated levels of traumatic stress experienced by professionals who have encountered suicide. Out of fear of more incidents and being held accountable, professionals increasingly turned to coercion to control and stop the suicidal behavior of adolescents (Farberow, 2005). The results suggest that due to high levels of distress after exposure to suicide, staff may develop dysfunctional action patterns resulting in a vicious coercive cycle: impact suicide → increases distress → fear for more incidents → increase in coercion → increases suicidal feelings of adolescents → increasing distress → fear for more incidents → and so forth. Roy et al. (2020) suggest that the prior usage of seclusion is a robust predictor of their future use as that use may provide, among other factors, a sense of control and competency (Mathieu & Geoffrion, 2022). Thus, psychological distress among professionals may negatively affect the subsequent care offered (Schwappach & Boluarte, 2009; Wurst et al., 2011). Remarkably, a few professionals did not report exacerbated use of coercion after experiencing suicide. These professionals described working in a team that enabled them to discuss fears, doubts, and uncertainties, negatively influencing the use of coercive measures in response to suicidal behavior. This finding is in line with Roy et al. (2020), who found that the more communication and openness among team members, the less coercive measures were used (Roy et al., 2020). Talking with colleagues in the aftermath of a completed suicide was found to be helpful in coping with the personal impact and strain resulting from exposure to suicide. At the same time, the professionals underlined that, however beneficial, collegial support often lasted too short. High staff turnover, unstable teams, high workload and a closed organizational culture impeded professionals from discussing the impact of suicide at work. Some reported that discussing the impact of suicide on professionals remained a taboo subject among colleagues, resulting in silencing of the issue. This is disturbing, as studies have demonstrated that stigma surrounding suicide leads to underestimating the psychological support needed and contributes to the distress experienced by clinicians (Gutin et al., 2011; Jordan et al., 2012). Moreover, the stigma of suicide among survivors may hinder the application of postvention interventions (Andriessen & Krysinska, 2012).

### Nonviolent resistance to prevent coercion

Parent training in nonviolent resistance has shown promising results in reducing suicide risk and distress in children, adolescents, and young adults (Omer & Dulberger, 2015). Adapting this approach to the context of SRYC can be beneficial for staff in dealing with the suicidal behavior among adolescents residing in SRYC. By training staff in nonviolent resistance, they can learn how to reduce the risk of potential and mutual distress surrounding suicidal threats, moving from helplessness to presence and from submission to resistance. This approach emphasizes self-control, care, and support, which are essential for reducing the suicide risk in adolescents. This method can be used even when the adolescent is not willing to cooperate, which is a common situation in SRYC. By using nonviolent resistance, staff can avoid relying on coercion, which has negative consequences for youth and professionals alike. By reducing the use of coercion, staff can prevent the above-mentioned vicious coercive cycle of increasing distress, fear of more incidents, and more coercion, which can ultimately lead to an increase in suicidal behavior among adolescents (Kaijadoe et al., 2023). Therefore, the use of nonviolent resistance can be an effective and humane approach for staff in SRYC to deal with the suicidal behavior of adolescents.

### Need for long-term postvention

The term postvention was first coined by Shneidman (1975), who used the word to describe the appropriate and helpful support after a terrible event (Shneidman, 1975). Postvention measures can lower emotional responses and traumatic impacts, and help professionals recover from the impact of a suicide (Erlich et al., 2017). Some professionals described how the lack of long-term postvention triggered escalating frustration levels. Moreover, due to dissatisfaction with the lack of support and postvention measures after a suicide, a few professionals decided to resign and leave the youth care sector. This is alarming, as the SRYC is already burdened with a vicious cycle of high staff turnover rates, major cutbacks, high rates of sick leave, and a shortage of trained personnel (Ministerie van Volksgezondheid Welzijn en Sport, 2019; Prismant, 2018). Concrete incidents in which entire SRYC teams fell apart after a suicide underline the potentially devastating impact of suicide on professionals (Huisman, 2020). Notably, some professionals reported an emotional response that resulted in PTSD. Skilled and dedicated support following an adolescent suicide is necessary to lower the emotional response and traumatic impact and to help professionals recover from the impact of suicide (Erlich et al., 2017).

### From blaming to a restorative culture

Notwithstanding, a postvention protocol alone is not enough to ensure that professionals receive the proper support when exposed to adolescent suicide (Dekker, 2012). Our results indicate that negative publicity in the (social) media and society, the review procedure of the Health and Youth Care Inspectorate, and the proclaimed aim of zero suicide seem to contribute to a culture of blame, which constrains professionals in many ways. Turner et al. (2020) advocate that health care practices should implement a restorative just culture alongside a zero-suicide framework (Turner et al., 2020). “Just culture” is a term used in health care to describe an organizational culture that encourages individuals to report errors, near misses, or system vulnerabilities without fear of punishment or retribution. Rather than blaming individuals, the focus is on learning from mistakes and improving the system to prevent similar errors from occurring in the future (Dekker, 2012; Marx, 2019). Although suicide is not an error, the principles of just culture can be applied to the system of secure residential youth care (SRYC). By applying a restorative just culture approach to SRYC, we can create an environment in which professionals feel supported to discuss fears and uncertainties in balancing safety and accountability when working with suicidal adolescents without fear of punishment or retribution. In addition, a restorative-just culture aims to repair the trust and relationships damaged after an incident. Restorative just culture principles are needed to replace backward-looking accountability with a more helpful focus on the hurts, needs, and obligations of all affected by suicide. Moreover, Turner et al. (2020) argue that implementing a zero-suicide framework may be compromised when it is not supported by such a substantial workplace cultural change (Turner et al., 2020).

We argue that the treatment of young people’s suicidal behavior requires the presence of and reflection on the actions of all those involved. The board of directors and managers should be careful that insecurities inherent in working with suicidal youth will not lead to paralysis or a “play it safe” culture. This requires vision, trust, contact, and proximity in an organizational culture. It is not the unwillingness of professionals in SRYC to opt for coercion. Professionals report a lack of alternatives for the use of coercion and too few well-trained staff members. Fear surrounds the subject, for what if it goes wrong? By discussing that fear with all involved (professionals, board of directors, management, parents, etc.) one makes connection and contact. There is a need for unconditional support for professionals in the aftermath of suicide. Hence, we have the following practical implications.

### Implications for practice

- The first big challenge for SRYC programs is to provide for an organizational culture in which professionals feel supported to discuss fear and uncertainty in balancing safety and accountability when working with suicidal adolescents.
- The second challenge for SRYC is proactively organizing postvention for professionals and teams exposed to suicide. The SRYC systems are responsible for caring for their professionals. Our results suggest that we are currently performing this infrequently and inadequately.
- Third, to reduce the use of coercive measures in response to suicidal behavior, we recommend that professionals in SRYC should be trained in alternative approaches. Non-violent resistance is a promising strategy that can be used to prevent coercion and suicide in SRYC. Despite its potential benefits, this approach has not yet been widely used in SRYC. Therefore, further research on this subject is required.
- Finally, there is a need for more (well-trained) personnel and increased knowledge on suicide prevention, with particular attention to the quality of the therapeutic alliance (Van Benthem et al., 2020). The use of experiential knowledge by lived experience experts may be a valuable additional (re)source to improve the care offered. However, this needs to be studied in greater detail.

### Recommendations for future research

Several questions remain to be answered to clarify the relationship between professional characteristics, the working climate, and the use of coercive measures. Further knowledge on this topic is needed to increase the effectiveness of training programs and organizational policies aimed at reducing coercive measures. Finally, in line with previous research, the first responders in this study described the emotional impact of suicide as traumatic (Lyra et al., 2021; Norton, 2017). No research has been conducted on strategies for managing psychological distress among professionals in SRYC who were first responders. More research into the subgroup of first responders in SRYC is needed to provide targeted support.

### Limitations and methodological considerations

Our results were based on 14 interviews with professionals from three SRYC organizations. Although this study provided detailed subjective reports from which new information could be drawn, the number of interviews did not allow for a straightforward generalization. Although the findings of this study are limited to a specific setting, namely SRYC in the Netherlands, they still hold relevance for residential care in general. This study provides detailed subjective reports on the impact of adolescent suicide on professionals. The novel information drawn from these reports can offer insights and a more comprehensive understanding of this issue. This understanding may be broadly applicable to professionals working in other residential care settings. For instance, the study sheds light on the emotional toll that suicidality can take on professionals. By understanding these impacts, residential care organizations can develop better support systems and resources for their staff, such as providing counseling or debriefing sessions after a suicide.

Given the subject’s sensitive nature, ample time was taken to become acquainted and establish a safe atmosphere before starting the interview. Suicidality is an impressive topic, and it is possible that professionals have not been able to provide a complete account of their experiences in an interview time of one to two hours. Furthermore, it is essential to consider that coding and working with qualitative data requires some form of interpretation. Although two researchers coded each interview independently to derive inter-coder reliability (Glaser & Strauss, 2008), and analyses were conducted with the research team, the results should be interpreted cautiously.

Considering these limitations, this study could be seen as an exploratory study with a first impression of the impact and effect of adolescent suicide on professionals in SRYC. These results can be used to expand future qualitative and quantitative research on this topic.

## Conclusion

Hopefully, this research will increase awareness among organizational settings and society as a whole that professionals in SRYC who are exposed to adolescent suicide are secondary victims. These professionals deserve our respect and must be better supported to keep up with their demanding work. The findings of this study suggest that a shortage of resources is one of the major issues encountered by SRYC professionals when handling adolescent suicides. The government must ensure that professionals in this field are equipped with the necessary resources to handle this demanding task. However, while additional resources are crucial, they alone do not provide a definitive solution to the challenges faced by SRYC. The availability of more resources must lead to improved working conditions, reduced workload, and implementation of a comprehensive suicide prevention training program. To truly enhance the quality of care to suicidal adolescents in SRYC, it is imperative to have skilled professionals who prioritize building meaningful relationships and avoiding coercion in response to suicidal behavior. Hence, it is essential to foster an attitude and culture within the organization that acknowledges the challenges surrounding suicide prevention, encourages collaboration at all levels, and ensures the necessary conditions for implementation in order for the field to attract and retain skilled professionals. The subject of adolescent suicide is characterized by fear and taboos. This tension can be addressed by implementing a just, safe culture in which professionals feel free to share their feelings, fears, and critical doubts. Trust may be the magical word. The high impact of suicide on professionals calls for leadership and organizational accountability. To put it in terms of Turner (p. 142): “the difference between a safe and an unsafe organization lies not in how many incidents it has, but in how the organization deals with the incidents” (Dekker, 2012).

## Supplementary Material

Supplemental Material
